# Imaging of Monoclonal Gammapathy of Undetermined Significance and Smoldering Multiple Myeloma

**DOI:** 10.3390/cancers12020486

**Published:** 2020-02-19

**Authors:** Bastien Jamet, Clément Bailly, Thomas Carlier, Cyrille Touzeau, Anne-Victoire Michaud, Mickael Bourgeois, Philippe Moreau, Caroline Bodet-Milin, Françoise Kraeber-Bodere

**Affiliations:** 1 Nuclear Medicine Unit, University Hospital, 44093 Nantes, France; bastien.jamet@chu-nantes.fr (B.J.); clement.bailly@chu-nantes.fr (C.B.); thomas.carlier@chu-nantes.fr (T.C.); annevictoire.michaud@chu-nantes.fr (A.-V.M.); mickael.bourgeois@univ-nantes.fr (M.B.); caroline.milin@chu-nantes.fr (C.B.-M.); 2 CRCINA, INSERM, CNRS, Angers University, Nantes University, 44093 Nantes, France; 3 Haematology Department, University Hospital, 44093 Nantes, France; cyrille.touzeau@chu-nantes.fr (C.T.); philippe.moreau@chu-nantes.fr (P.M.); 4 Nuclear Medicine Unit, ICO-Gauducheau, 44805 Nantes-Saint-Herblain, France

**Keywords:** MGUS, SMM, imaging, WBCT, MRI, FDG-PET/CT

## Abstract

Multiple myeloma (MM) is always preceded by an initial monoclonal gammopathy of undetermined significance (MGUS) that then develops into asymptomatic or smoldering multiple myeloma (SMM), which constitutes an intermediate clinical stage between MGUS and MM. According to a recent study, risk factors for faster MGUS to MM progression include an M protein of 1.5 g/dL or more and an abnormal free light chain ratio in patients with non-IgM MGUS. Therefore, the International Myeloma Working Group (IMWG) decided to recommend whole-body computed tomography (WBCT) for patients with high-risk MGUS in order to exclude early bone destruction. Studies evaluating magnetic resonance imaging (MRI) in SMM found an optimal cutoff of two or more focal lesions to be of prognostic significance for fast progression into symptomatic disease and considered this biomarker as a myeloma-defining event (MDE) needing to start therapy with the aim to avoid progression to harmful bone lesions. Moreover, studies assessing positron emission tomography (PET) with computed tomography (CT) using 18F-deoxyglucose (FDG) (FDG-PET/CT) in SMM showed that presence of focal bone lesion without underlying osteolysis is associated with a rapid progression to symptomatic MM. Latest IMWG guidelines recommended to perform WBCT (either CT alone or as part of an FDG-PET/CT protocol) as the first imaging technique at suspected SMM and, if these images are negative or inconclusive, to perform whole-body MRI. The goal of this paper is to clarify the role of different imaging modalities in MGUS and SMM workups.

## 1. Introduction

Multiple myeloma (MM) is a hematological neoplasm characterized by the clonal proliferation of malignant plasma cells, primarily in the bone marrow. It is always preceded by an initial monoclonal gammopathy of undetermined significance (MGUS) that then develops into asymptomatic or smoldering multiple myeloma (SMM), which constitutes an intermediate clinical stage between MGUS and MM [1]. Rate of progression from MGUS and SMM to MM are 0.5–1% per year and 10% per year, respectively, for the first 5 years, with thresholds of serum monoclonal protein and bone marrow plasmacytosis differing between both classifications. 

Main presenting symptoms of MM are included in the CRAB acronym as hypercalcemia (C), renal impairment (R), anemia (A) and bone disease (B), with bone disease being the more frequent symptom of MM, affecting more than 80% of all patients involved [2]. Thus, imaging plays a crucial role in detecting bone damage, especially in early stages of disease, with the aim of not delaying the initiation of treatment and avoiding progression to harmful bone lesions. The goal of this paper is to clarify the role of different imaging modalities in MGUS and SMM workups.

## 2. Imaging of Monoclonal Gammopathy of Undetermined Significance

MGUS is defined by the presence of less than 10% of monoclonal plasma cells in the bone marrow, along with the presence of monoclonal protein in serum or urine, or both [3]. It has a high incidence of 3.2% in individuals of 50 years of age or older and of 5.3% in persons of 70 years or older [4].

Recently updated analysis of the large southeastern Minnesota cohort [5] concerning 1384 patients with MGUS with a median follow-up of 34 years showed among patients with non-IgM MGUS that the presence of two adverse risk factors—namely, an abnormal serum free light chain ratio (ratio of kappa to lambda free light chains) and a high serum monoclonal protein (M protein) level (≥1.5 g per deciliter)—was associated with a risk of progression to active MM at 20 years of 30%, as compared with 20% among those who had only one risk factor and 7% among those who had neither risk factor. Significant differences were noted in the risk of progression to active MM between patients with the same non-IgM MGUS; therefore, it is questionable to perform whole-body (WB) imaging in patients with high-risk MGUS to exclude bone damage.

Several studies have previously assessed whole-body magnetic resonance imaging (WBMRI) at MGUS diagnosis. A study analyzing the findings of WBMRI (conventional, without diffusion-weighted images) in 137 consecutive patients with MGUS in 2014 showed bone focal lesions (FL) in 23.4% of patients. Presence and number of FL as well as value of the M protein were independently predictive of progression into a symptomatic disease requiring systemic treatment [6]. In a smaller cohort of 29 individuals with MGUS assessed by conventional WBMRI, only one (3%) patient had FL [7]. Moreover, the relatively new functional dynamic contrast-enhanced magnetic resonance imaging (DCE-MRI) and its derived parameters, reflecting bone marrow angiogenesis/microcirculation, which plays a crucial role in the pathogenesis of MM, have also been assessed in a study with 33 healthy controls and patients with MGUS (*n* = 69) or SMM (*n* = 79). DCE-MRI-derived parameters failed to predict progression to active MM in individuals with MGUS, conversely to patients with SMM [8].

Unfortunately, there is no prospective assessment available of whole-body computed tomography (WBCT) at MGUS diagnosis, especially in the group of patients with one or two adverse risk factors. In the same way, to date, there are no published data about the potential role of positron emission tomography (PET) with CT using 18F-deoxyglucose (FDG) (FDG-PET/CT) in patients with MGUS.

Despite the lack of data available regardingimaging assessment in this setting, IMWG has recently recommended [9] to perform WBCT in suspected high-risk non-IgM MGUS to rule out MM, considering the benefits of WBCT to exclude bone destruction, the most important symptom to be excluded in patients with MGUS. IMWG also recommended for patients with equivocal findings on WBCT to perform complementary WBMRI (Level IV) and to not perform follow-up bone imaging if WBCT is negative unless there are clinical/biological signs of progression to symptomatic disease. 

## 3. Imaging of Smoldering Multiple Myeloma

SMM is defined by the presence of more than 10% of monoclonal plasma cells in the bone marrow, along with the presence of monoclonal protein in serum or urine, or both [3], without CRAB symptoms. SMM is a heterogeneous definition including patients with a very slow (several years) progression to active MM (low-risk SMM) and patients progressing very rapidly to symptomatic MM in less than 2 years (high-risk SMM). Consequently, the definition of symptomatic MM, a clinical stage requiring treatment, was revised in 2014 by the IMWG, integrating new pejorative prognostic biomarkers [3] as myeloma-defining events (MDE), in addition to usual CRAB MDE, with the aim of not delaying the initiation of treatment for patients classified as high-risk SMM in order to avoid progression to harmful bone lesions or renal insufficiency. Based notably on large Mayo Clinic [10] and Larsen et al. [11] studies, clonal bone marrow plasma cell percentage ≥60% and involved/uninvolved serum free light chain ratio ≥100 were respectively included as MDE, predicting at least a rate of 80% of progression to MM within 2 years. 

**Figure 1:**
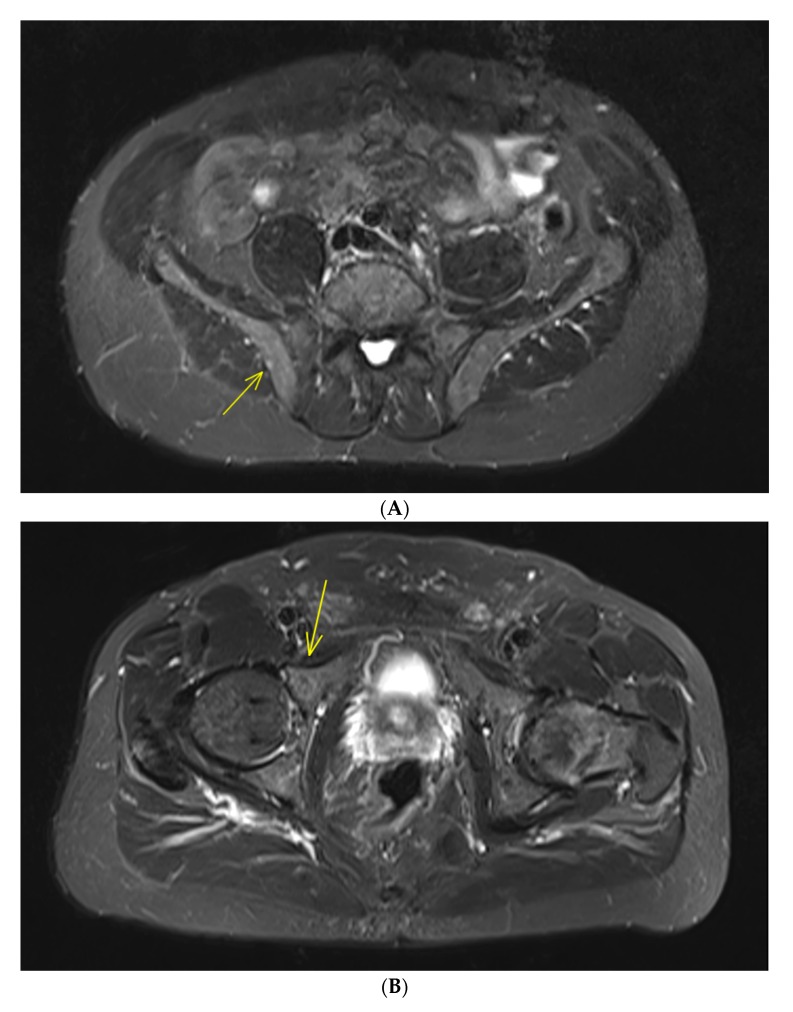

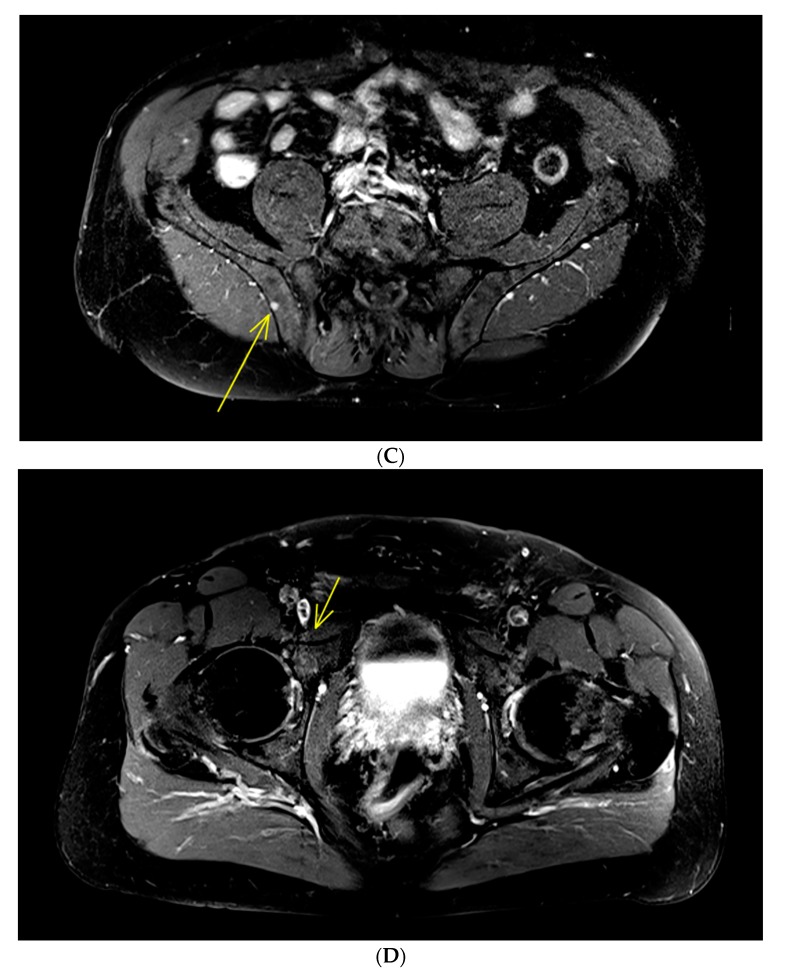

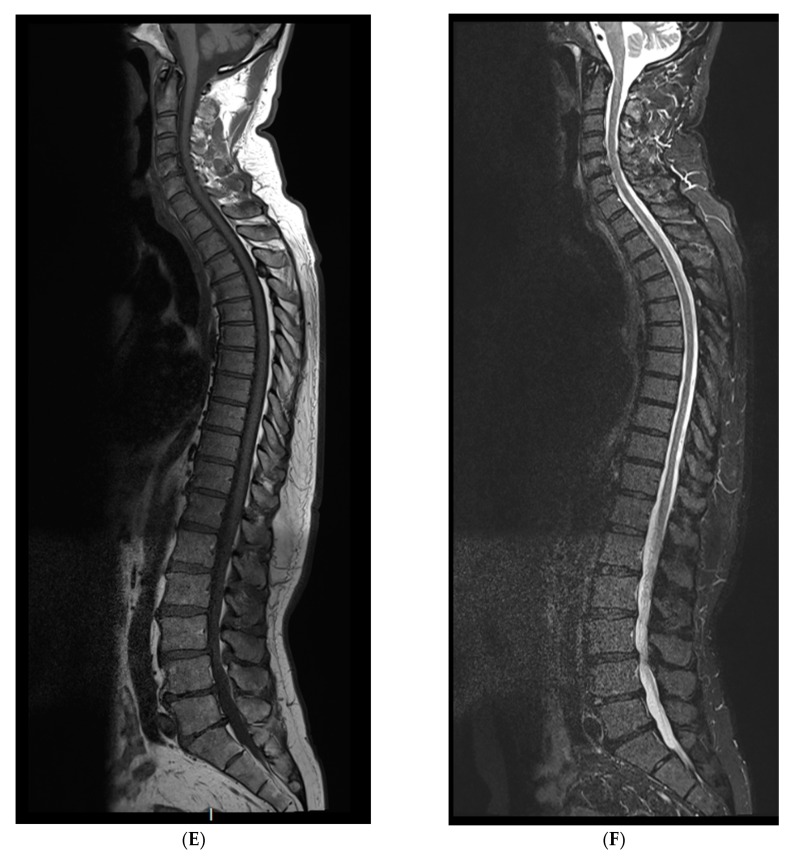
IgGk smoldering multiple myeloma (SMM) patient with negative whole-body computed tomography. Magnetic resonance imaging of the rachis and pelvis shows two focal bone lesions with increased signal intensity in T2-weighted STIR (short-tau inversion recovery) images. One small lesion (diameter: 5 mm) of the right iliac crest (**A**) and one bigger lesion (diameter: 14 mm) of the right acetabulum anterior column (**B**), with corresponding enhancement after Gadolinium injection in T1-weighted images with fat saturation (**C**,**D**). No further focal lesion of the rachis was detected in whole-rachis sagittal T1-weighted and T2-weighted STIR sequences (**E**,**F**). These two focal bone lesions are considered a myeloma-defining event leading to the recommendation to treat this patient.

Studies relating to conventional magnetic resonance imaging (MRI) in patients with SMM have also shown presence of more than 1 FL (with size > 5 mm) was predicting of rapid progression to active MM, leading to inclusion of this biomarker as MDE (Figure 1). Indeed, in a large cohort of 149 SMM patients assessed by conventional WBMRI, presence of more than one FL was a strong adverse prognostic factor for progression into symptomatic MM (more than 60% at 2 years) in multivariate analysis, as well as diffuse bone marrow infiltration pattern [12]. These results have been confirmed in a smaller study of 67 SMM patients assessed by spinal MRI [13]. For patients with more than one FL, the 1-year progression to symptomatic MM rate was 44%, the 2-year progression rate was 69% and the 3-year progression rate was 85%, versus 1-, 2- and 3-year progression rates for those with no FL of 8%, 13% and 22%, respectively.

In a research setting, relatively new functional MRI approaches have showed promising results for disease detection, prediction of outcome and assessment of response to therapy in MM, shedding new light on MM imaging, especially in addition to FDG-PET/CT. In SMM, a study assessing DCE-MRI (reflecting bone marrow angiogenesis/microcirculation) in a cohort of 79 SMM patients [8] showed prognostic significance of DCE-MRI-derived parameters median of amplitude A (associated with blood volume) and exchange rate constant kep (surrogate for vessel permeability and perfusion) for time to progression to MM. Patients with amplitude A above the optimal cutoff point of 0.89 arbitrary units had a 2-year progression rate into symptomatic disease of 80%. Another study [14] confirmed the ability of DCE-MRI (by the wash-in perfusion parameter) to predict time to progression to symptomatic MM and to differentiate SMM to symptomatic MM patients. Moreover, DCE-MRI perfusion parameter was positively correlated with the adverse prognostic feature of angiogenesis–angiopoietin-1 (Angp-1/Angp-2) ratio (reduced in symptomatic MM compared to SMM and MGUS patients).

**Figure 2:**
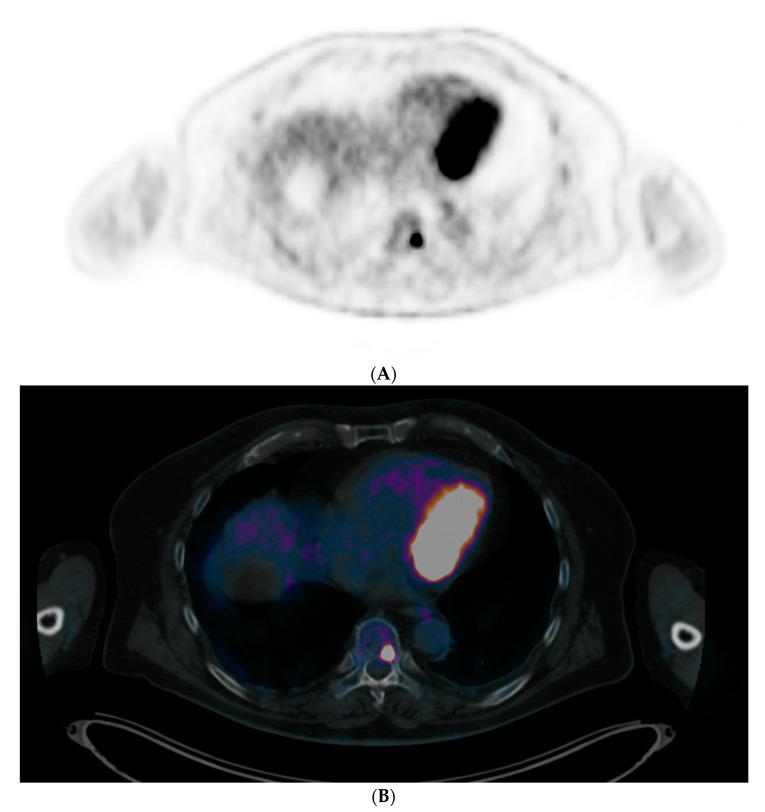

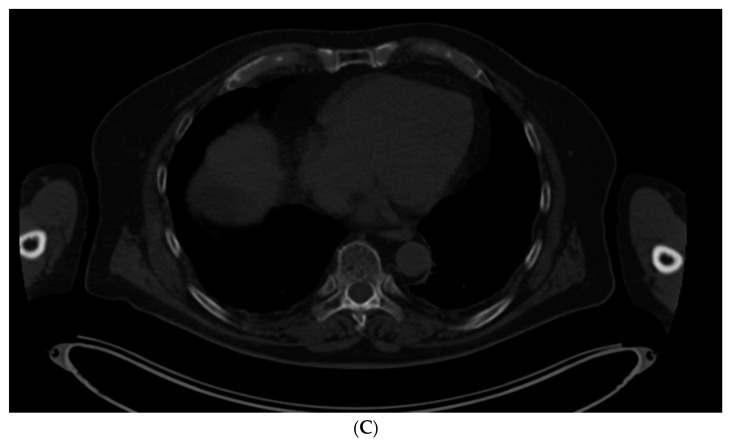
IgAk SMM patient with only one focal lesion of a thoracic vertebral body (maximum standardized uptake value (SUVmax): 15.34) depicted in FDG-PET/CT. (**A**: Positron emission tomography (PET) slice and **B**: PET/CT fused slice). Because of the absence of osteolytic lesions on the opposite CT (**C**), biopsy was performed in this area, revealing a focal monotype k accumulation of clonal plasma cells, upgrading the patient to symptomatic multiple myeloma (MM) requiring treatment.

FDG-PET/CT imaging has also proved its utility in SMM setting, showing prognostic value. Even if the latest updated MM IMWG definitions [3] indicated that osteolysis on CT was mandatory when an FL is depicted by FDG-PET/CT for considering it as a criterion for starting therapy at MM initial diagnosis, all prospective studies since 2009 defined FL as foci of uptake with or without osteolysis because metabolic could precede morphological abnormalities (Figure 2).

In SMM, a positive FDG-PET/CT defined by the presence of FL without underlying osteolytic lesions is associated with a rapid progression to symptomatic MM. Indeed, in a cohort of 122 SMM patients assessed by FDG-PET/CT, the probability of progression to symptomatic MM without therapy within 2 years for positive FDG-PET/CT patients was 75% versus 30% for patients with a negative PET [15]. It should be clarified that of 25 patients with a positive FDG-PET/CT, the probability of progression was 87% at 2 years in those with evidence of underlying osteolysis (*n* = 16) and 61% in patients without evidence of osteolysis (*n* = 9). In another prospective study of 120 SMM patients, the Bologna Group [16] reported a similar rate of progression to symptomatic MM at 2 years of 58% for patients with positive PET (all without evidence of underlying osteolysis) versus 33% for patients with a negative PET. These FDG-PET/CT results have been published after the latest IMWG MM updated definitions [3] and thus are not yet considered as an MDE leading to the recommendation to treat these patients.

The recent large IMWG’ study [17] confirming superiority of WBCT over conventional skeletal survey (CSS) for bone disease detection in MM also related to SMM. Of 66 patients with SMM based on CSS, 12 (22.2%) had osteolytic lesions on WBCT. Patients with lytic bone lesions in WBCT had a median time to progression of 38 months versus 82 months for those without bone destruction. These results (compatible with earlier and much smaller studies [18,19,20,21]) led to 2019 IMWG’ guidelines to perform WBCT (either CT alone or as part of an FDG-PET/CT protocol) as the first imaging technique in SMM (as in symptomatic MM, Level III). If WBCT is negative, WBMRI must be performed (Level IV) and, if it is negative, patients should be monitored with a yearly follow-up by WBMRI. If only one FL is depicted by WBMRI, it is possible to consider an oversight every 6 months to track increase in size, number or FL.

## 4. Perspectives

At non-IgM MGUS diagnosis, it is henceforth known that patients do not have the same risks of progression to active MM according to presence (or absence) of two adverse biological risk factors. IMWG recommended to perform WBCT for suspected high-risk non-IgM MGUS to rule out bone destruction despite lack of data published. Highly sensitive functional imaging techniques such as FDG-PET/CT or diffusion-weighted magnetic resonance imaging (DW-MRI), reflecting differences in motion of water at a cellular level in tissues with the apparent diffusion coefficient (ADC), in subsets of patients with one or two adverse risk factors for early disease detection or prediction of progression to active MM, might be attempted. 

In the same way, with the aim to include a positive PET (defined by the presence of an FL without underlying osteolytic lesions) as an MDE or to improve definition of an FL by MRI, one may consider adding concepts of restriction of water diffusion (high signal on source diffusion images and low ADC) to morphological abnormalities. Prospective comparisons of FDG-PET and DW/DCE-MRI (simultaneously acquired with a PET-MRI system) versus WBCT for very precocious disease detection at SMM diagnosis could be interesting, keeping in mind disease burden and bone marrow infiltration are usually lower in this early phase of the disease.

## 5. Conclusions

Highly sensitive imaging techniques are very important in SMM and MGUS early phases of the MM disease, especially in patients with high risk of progression to symptomatic disease. Despite the lack of data available, IMWG has recently recommended to perform WBCT in suspected high-risk non-IgM MGUS to rule out bone destruction. In SMM, it is recommended to perform WBCT (either CT alone or as part of an FDG-PET/CT protocol) as the first imaging technique choice in order to exclude osteolytic FL, and, if negative, to perform WBMRI imaging in search of MDE. Additional data provided by FDG-PET and new functional MRI approaches such as DW/DCE-MRI, simultaneously acquired with a PET-MRI system, should be investigated in MGUS and SMM settings for premature detection of bone disease and prediction of outcome.
